# Clozapine, relapse, and adverse events: a 10-year electronic cohort study in Canada

**DOI:** 10.1192/bjp.2024.140

**Published:** 2024-12

**Authors:** Lloyd Balbuena, Shawn Halayka, Andrew Lee, A.G. Ahmed, Tamara Hinz, Nathan Kolla, Jenna Pylypow

**Affiliations:** Department of Psychiatry, University of Saskatchewan, Canada; College of Medicine, University of Saskatchewan, Canada; Division of Child and Adolescent Psychiatry, University of Saskatchewan, Canada

**Keywords:** Psychotic disorders/schizophrenia, register-based study, antipsychotics, drug or substance interactions and side effects

## Abstract

**Background:**

Clozapine is the most effective medication for treatment-resistant psychoses, but the balance of benefits and risks is understudied in real-world settings.

**Aims:**

To examine the relative re-hospitalisation rates for mental health relapse and adverse events associated with clozapine and other antipsychotics in adult and child/youth cohorts.

**Method:**

Data were obtained from the Canadian Institute of Health Information for adults (*n* = 45 616) and children/youth (*n* = 1476) initially hospitalised for mental health conditions in British Columbia, Manitoba and Saskatchewan from 2008 to 2018. Patient demographics and hospitalisations were linked with antipsychotic prescriptions dispensed following the initial visit. Recurrent events survival analysis for relapse and adverse events were created and compared between clozapine and other antipsychotics.

**Results:**

In adults, clozapine was associated with a 14% lower relapse rate versus other drugs (adjusted hazard ratio: 0.86, 95% CI: 0.83–0.90) over the 10-year follow-up. In the first 21 months, the relapse rate was higher for clozapine but then reversed. Over 1000 person-months, clozapine-treated adults could be expected to have 38 relapse hospitalisations compared with 45 for other drugs. In children/youth, clozapine had a 38% lower relapse rate compared with other antipsychotic medications (adjusted hazard ratio: 0.62, 95% CI: 0.49–0.78) over the follow-up period. This equates to 29 hospitalisations for clozapine and 48 for other drugs over 1000 person-months. In adults, clozapine had a higher risk for adverse events (hazard ratio: 1.34, 95% CI: 1.18–1.54) over the entire follow-up compared with other antipsychotics. This equates to 1.77 and 1.30 hospitalisations over 1000 person-months for clozapine and other drugs, respectively.

**Conclusions:**

Clozapine was associated with lower relapse overall, but this was accompanied by higher adverse events for adults. For children/youth, clozapine was associated with lower relapse all throughout and had no difference in adverse events compared with other antipsychotics.

## Adult patients

Clozapine is the most effective drug for treatment-resistant schizophrenia in adults. The CUtLASS 2 and phase 2E CATIE studies have shown that clozapine is more effective than quetiapine and risperidone, and more effective than switching to another second-generation antipsychotic (SGA) when there is an inadequate therapeutic response.^1,2^ Several treatment guidelines across the world recommend clozapine as the gold standard for difficult-to-treat cases of adult schizophrenia^3,4^ and treatment-resistant bipolar disorder.^5^ Compared with first-generation antipsychotics, risperidone and quetiapine,^6,7^ clozapine is associated with lower mortality and re-hospitalisation for schizophrenia.^8^

## Child/youth patients

In contrast to the solid body of evidence for its efficacy in adults, the role of clozapine in early-onset schizophrenia (EOS) remains less understood. There is a lack of robust double-blinded studies in children/youth, primarily due to the rarity of the condition. However, an emerging body of evidence supports the use of clozapine in children with refractory cases of EOS. Small, double-blind trials of clozapine versus haloperidol and olanzapine, and clozapine versus olanzapine in EOS demonstrated superior efficacy of clozapine.^9,10^ The Canadian Schizophrenia Guidelines and the British National Institute for Health Care Excellence (NICE) guidelines recommend offering clozapine to children with schizophrenia spectrum illness who have not responded adequately to at least two different antipsychotics administered for 6 to 8 weeks each.^11^

## Real-world outcomes

Real-world data about mental health re-hospitalisations for clozapine and other psychotropic medications are a valuable source of complementary evidence. As a result of their study design, randomised clinical trials provide limited evidence for long-term outcomes, so in this regard, real-world data can shed more light on more long-term outcomes. About 1% of patients on clozapine develop agranulocytosis, and about 3% have mild to moderate neutropenia.^12^ Clozapine induces cardiometabolic side effects, such as weight gain and obesity,^13^ but this also applies to olanzapine and to a lesser extent other SGAs.^14^ Myocarditis has been observed in clozapine-treated patients.^15^ A Taiwanese study reported that schizophrenia patients on clozapine were more likely to develop pneumonia 30 days after an upper respiratory tract infection.^16^ Clozapine may cause swallowing difficulties leading to aspiration, which facilitates the movement of pathogens from the upper to the lower respiratory tract.^16^

Among children, adverse effects for clozapine have also been reported. Fleischhaker and colleagues^17^ found that among 45 youths aged 9 to 21 years treated for 45 weeks with olanzapine, risperidone or clozapine, the average weight gain associated with clozapine was 9.5 ± 10.4 kg, comparable with risperidone (7.2 ± 5.3 kg) and olanzapine (16.8 ± 8.8 kg). Hypertriglyceridemia was found to occur in about 8–22% of clozapine-treated EOS patients with emergent diabetes occurring in about 6% of clozapine-treated youth^9^ versus a type 2 diabetes incidence rate of three cases per 1000 patient-years for all youth treated with antipsychotics.^18^ A retrospective chart review of 172 clozapine-treated children and adolescents reported the one-year prevalence of neutropenia as 13%.^19^ In contrast, a meta-analysis of 20 studies did not find an association between clozapine and neutropenia risk.^20^

The objective of the present study was to perform a risk-benefit evaluation of clozapine in a cohort of adults and children from three Canadian provinces. The risk component was examined by comparing adverse event rates in clozapine with those of other antipsychotics. The benefit component was examined by comparing mental health-related hospitalisations by drug group.

## Method

### Data and participants

The present study used hospitalisation and drug dispensing data obtained from the Canadian Institute of Health Information (CIHI). We created separate electronic cohorts of adults and children/youth. The cohorts were patients from British Columbia, Manitoba and Saskatchewan who were diagnosed with schizophrenia, schizoaffective disorder, bipolar disorder and other psychoses at their initial hospital visit, possibly comorbid with other disorders. For the children, common childhood-onset comorbid disorders were also recorded: attention-deficit hyperactivity disorder (ADHD), oppositional defiant disorder (ODD), conduct disorder and autism. Other diagnoses (e.g. personality disorders) were also searched for both cohorts but these had very low frequency. The three provinces were chosen because they had complete linkage of hospitalisation and drug use data.

The cohorts were followed over a maximum of 10 years from 2008 to 2018, and each person had hospitalisation records for ambulatory visits (from the National Ambulatory Care Reporting System) and inpatient stays (Discharge Abstract Database). CIHI provided records of psychotropic medications obtained from the National Prescription Drug Utilization Information System. The drug data did not contain dose information, so it was not possible to tell if the medications met or exceeded the minimum effective dose. For all medications, the quantity of the medication dispensed was unavailable, so it was assumed that whenever a prescription was filled, the patient had one month's supply – a typical practice in Canadian pharmacies.

The adults had a mean follow-up of 62 months (median: 59) and 54 months (median: 49) for children/youth. After consolidating hospitalisation and medication records, a chronology of hospitalisations and medications were created for each patient. Table 1 illustrates raw data for a fictional patient.

**Table 1:** Historical record for a fictional patient

Age at index	Clozapine	Start	End	Adverse event	Mental health relapse	Event (spell type)
17	0	1 Jun 2010	15 Jun 2010	0	0	Initial hospitalisation
17	0	26 Jun 2010	24 Oct 2010	0	0	Quetiapine
17	0	25 Oct 2010	5 Mar 2011	0	0	Quetiapine
17	0	9 Apr 2011	4 Jun 2011	0	0	Quetiapine
17	0	5 Jun 2011	13 Jun 2011	0	1	Mental health re-hospitalisation
17	1	14 Jun 2011	17 Jul 2011	0	0	Clozapine
17	1	18 Jul 2011	1 Aug 2011	1	0	Adverse event re-hospitalisation
17	0	2 Aug 2011	5 Dec 2011	0	0	Olanzapine
17	0	8 Jan 2012	28 Jan 2012	0	0	Olanzapine
17	0	29 Jan 2012	2 Feb 2012	0	1	Mental health re-hospitalisation
17	0	3 Feb 2012	11 Mar 2012	0	0	Olanzapine
17	N/A	12 May 2012	14 May 2012	0	1	Mental health re-hospitalisation

### Variables of interest

The primary outcome was mental health visits or admissions after being discharged from the initial visit (i.e. relapses). The patient in Table 1, for example, had three mental health re-hospitalisations over the follow-up period – coded as ‘1’ in the column MH [mental health] relapse. The secondary outcome was adverse events, defined as neutropenia (agranulocytosis, D70), cardiomyopathy (I42–I49), myocarditis (I40, I41, I51), pneumonia (J12–J18) and constipation (K59). A binary variable was created that had the value 1 if the patient was hospitalised for any of these codes, and 0 otherwise. The fictional patient in Table 1 had one adverse event re-hospitalisation occurring after clozapine. When there was a gap of 30 days or more between a medication spell and hospitalisation, the clozapine variable was set to missing (Table 1, last row). In effect, records such as these would not be linked with any drug and were excluded from the analysis.

Medication status (clozapine or other drug) was the main predictor of interest. The ‘other drug’ category consisted of first and second-generation antipsychotics as well as other psychotropic medications. The SGAs were the following: aripiprazole, asenapine, lurasidone, olanzapine, quetiapine, risperidone, paliperidone and ziprasidone. The first-generation antipsychotics were the following: chlorpromazine, fluphenazine, haloperidol, loxapine, perphenazine, pimozide, thiotexine and trifluoperazine. Other psychotropic medications (e.g. lithium) were also recorded.

Socio-demographic variables including sex, age at index hospitalisation, and urban or non-urban residence were entered as covariates in our models. Unlike the sociodemographic variables whose values were fixed to those in the initial visit, medication status, adverse events and relapses varied during the follow-up.

### Creation of medication and hospitalisation spells

In general, patients had as many rows as they had records of medication refills and re-hospitalisations for up to 10 years of follow-up. To facilitate the analysis, spells were created, which are defined as consecutive periods of medication or hospitalisation. For example, the second and third rows of Table 1 were later merged to a single row. Hospitalisation episodes were merged into a spell only if they occurred within 3 days of each other.

For the analysis, the various spells were classified into one of four categories: adverse event, mental health re-hospitalisation, other drug and clozapine. In some cases, there were overlapping spells of medication and hospitalisation, as might occur when a person has a month's supply of medication but is admitted to the hospital for an acute mental episode. The overlap was resolved by assigning a priority ranking among spells: adverse events, then mental health hospitalisations, and finally clozapine and other drugs with lower priority. Spells were merged and ranked using the Newspell package^21^ in Stata.

When a patient was on several antipsychotics simultaneously (i.e. polypharmacy) ‘spell’ was categorised as other drug unless they were also on clozapine. The assignment of medications to spells that have temporal sequence and duration allows inference about their effect on dependent variables.

The authors assert that all procedures contributing to this work comply with the ethical standards of the relevant national and institutional committees on human experimentation and with the Declaration of Helsinki of 1975, as revised in 2013. All procedures involving human subjects/patients were approved by the University of Saskatchewan Research Ethics Board (Bio-563). As the study used anonymised data provided by CIHI, obtaining consent was not necessary.

### Analysis

Adult and child cohorts were analysed separately by fitting Royston–Parmar survival models for recurrent events. Briefly, Royston–Parmar models relate the cumulative hazard to survival as a monotonic increasing function of time.^22^ Royston–Parmar is a parametric technique that uses splines, with a varying number of knots to model how the baseline cumulative hazard changes with time. One can think of a spline as a tight rope that is given some slack, and the knots are where the rope curls to follow the shape of the data.

For both adults and children/youth, separate models were fitted for mental health relapses and adverse events – a total of four survival models. Proportional cumulative hazards models (with log of time scale) were created and the number of knots for the splines based on Akaike information criterion (AIC) and Bayesian information criterion (BIC) goodness of fit statistics were selected. Time-varying coefficients were entered when necessary, to allow for non-proportional hazard ratios. These were implemented in the STPM3^23^ Stata package.

After fitting each model, population averaged survival probabilities of patients were calculated by drug group. These survival curves were adjusted for sex, age at index hospitalisation and urban/non-urban residence – in other words, differences in survival probability are attributable only to drug status and not to differences in the covariates.^24^ Standardised survival probabilities also go beyond the usual hazard ratios, because they do not invoke the proportionality assumption.^24^

The code used for implementing (reproducing) the analyses is publicly available at https://github.com/lloydxeno/antipsychotics and at https://github.com/sjhalayka/dad_nacrs.

## Results

A total of 56 568 adults and 2159 children/youth met the eligibility criteria at the index hospitalisation. However, 10 952 adults and 683 children/youth relapsed or were hospitalised for an adverse event before an antipsychotic was dispensed. Excluding these groups left an analysis sample of 45 616 adults and 1476 children/youth. The adults were between 18 and 106 years old at their index visit, 52% were women and 17% lived in non-urban areas. The children/youth were between 7 and 17 years old at the index visit, 47% were girls and 16% lived in non-urban areas (Table 2).

**Table 2:** Demographic characteristics at index hospitalisation

Variable	Child/youth (%)	Adult (%)
*n*	1476	45 616
Mean age at index hospital visit (s.d.)	15.35 (1.89)	44.84 (17.21)
Women	766 (51.90)	21 658 (47.48)
Province		
British Columbia	1100 (74.53)	31 983 (70.11)
Manitoba	139 (9.42)	7326 (16.06)
Saskatchewan	237 (16.06)	6307 (13.83)
Diagnoses		
Schizophrenia	398 (26.96)	18 259 (40.03)
Schizoaffective disorder	86 (5.83)	6240 (13.68)
Bipolar disorder	1060 (71.82)	22 254 (48.79)
Other psychosis	167 (11.31)	2083 (4.57)
ADHD	202 (13.69)	382 (0.84)
Conduct disorder	0 (0.00)	5 (0.01)
ODD	60 (4.07)	31 (0.07)
Autism	57 (3.86)	118 (0.26)
Residence		
Urban	1237 (83.81)	37 779 (82.82)
Rural/other/unknown	239 (16.19)	7837 (17.18)

ADHD, attention-deficit hyperactivity disorder; ODD, oppositional defiant disorder.

### Adult cohort

**Fig. 1:**
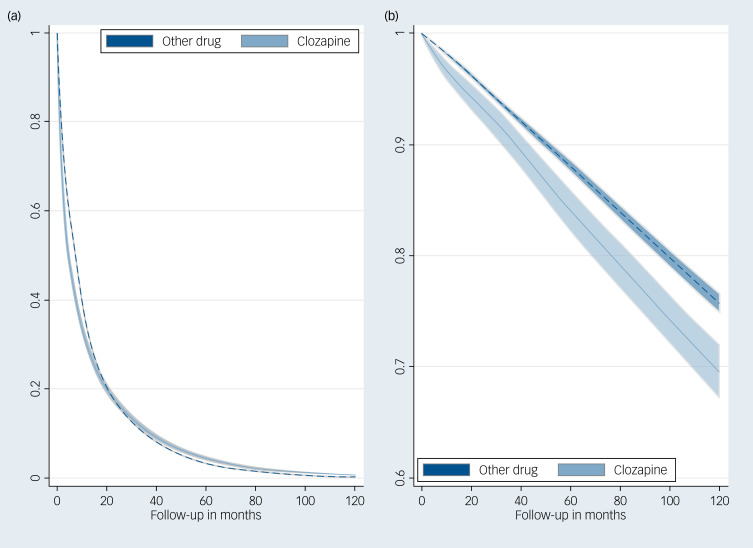
Survival curves for (a) relapse and (b) adverse events in adult patients from British Columbia, Manitoba and Saskatchewan.

There were 176 691 mental health relapses over the follow-up period. Clozapine was associated with a 14% lower rate of re-hospitalisations compared with other drugs (hazard ratio: 0.86, 95% CI: 0.83–0.90) adjusted for sex, age at index hospitalisation and residence. However, the hazard ratio was time-dependent: clozapine had a higher re-hospitalisation rate (*B* = 2.11, 95% CI: 1.78–2.44) earlier in the follow-up (i.e. up to 21 months) and a lower one thereafter (i.e. higher survival probability) (see Fig. 1a and Supplementary Table 1 available at http://doi.org/10.1192/bjp.2024.140) Over 1000 person-months, the expected number of relapse hospitalisations for clozapine-treated adults is 38 (95% CI: 35–41) *v.* 45 (95% CI: 43–47) for other drugs. Clozapine treatment was associated with 4 fewer hospitalisation days of relapse per person over 120 months (52 days for clozapine *v.* 56 days for other drugs).

There were 11 405 adverse events resulting in re-hospitalisation over the follow-up (Table 3) (see Fig. 1b). Clozapine had a higher risk for adverse events (hazard ratio: 1.34, 95% CI: 1.18–1.54) over the entire follow-up compared with other antipsychotics (see Supplementary Table 2). Over 1000 person-months, the expected number of adverse event hospitalisations for clozapine-treated adults is 1.77 (95% CI: 1.40–2.24) *v.* 1.30 (95% CI: 1.15–1.48) for other drugs.

**Table 3:** Adverse event hospitalisations

	Children (*n* = 1476)		Adults (45 616)	
	Incidents	*n* (%)	Incidents	*n* (%)
Constipation	53	35 (2.37)	2502	1621 (3.55)
Pneumonia	54	46 (3.12)	8319	4521 (9.91)
Neutropenia	3	3 (0.20)	284	223 (0.49)
Cardiomyopathy	1	1 (0.07)	228	158 (0.35)
Myocarditis	3	2 (0.14)	72	64 (0.14)

### Child/youth cohort

There were 5417 new mental health relapses among children and youth. In contrast to the relapse model in the adult cohort, clozapine treatment was associated with lower relapse throughout the entire follow up.

**Fig. 2:**
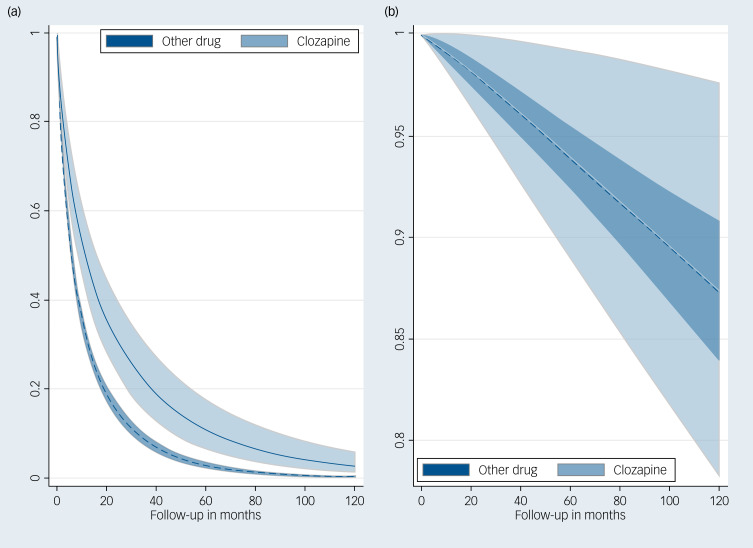
Survival curves for (a) relapse and (b) adverse events in child/youth patients from British Columbia, Manitoba and Saskatchewan.

Clozapine had a 38% lower relapse rate compared with other antipsychotic medications (adjusted hazard ratio: 0.62, 95% CI: 0.49–0.78) over the follow-up period, adjusted for sex, age at index hospitalisation and residence (see Fig. 2a and Supplementary Table 3). Over 1000 person-months, the expected number of relapse hospitalisations for clozapine-treated children/youth is 29 (95% CI: 22–39) *v.* 48 (95% CI: 40–57) for other drugs. Over 1000 person-months, the expected number of adverse event hospitalisations in children/youth did not differ between clozapine and other drugs (both 1.25).

There were 114 hospitalisations for adverse events over the follow-up period (Table 3). There was no difference in adverse event rates by drug group (hazard ratio: 0.99, 95% CI: 0.40–2.47), which probably resulted from the very small number of adverse events (see Fig. 2b and Supplementary Table 4).

## Discussion

In this study, an electronic cohort of patients from three Canadian provinces was analysed and hazards and survival probabilities were calculated for mental health relapse and adverse events over a 10-year follow-up. There were two main findings. First, in the adult cohort, clozapine initially had a higher risk for mental health relapse (up to 21 months), a trend that reversed over the long run. Adults on clozapine had a higher incidence of adverse events but had seven fewer relapse hospitalisations while conceding less than one adverse hospitalisation to other drugs. Second, in the children's cohort, clozapine had a lower risk of mental health relapse versus other antipsychotics throughout the follow-up period, while the incidence of adverse events did not differ.

In the adult cohort, the inflection point (in which clozapine's outcomes became more favourable) occurred at 21 months. Without further information about illness severity and factors affecting treatment, it is difficult to interpret clozapine's efficacy lag. One possibility is that it reflects natural variation in patient response and tolerance of clozapine. In a small study of 32 schizophrenia patients treated with clozapine and followed up to 1 year, the responders did so at 1, 2, 3 and 6 months.^25^ Nine patients (28%) never responded. Another study reported that adverse reactions to clozapine are most pronounced in the first few months – during this time up to 45% discontinue clozapine treatment.^26^ The reasons for discontinuing included dislike of monitoring, beliefs that treatment is unnecessary and physicians’ decisions. Altogether, these findings suggest that the worse relapse rate for clozapine in the short-term could be attributable to the mix of responders and non-responders, compliant and non-compliant patients during this period. The patients still on clozapine after the inflection point are more likely to have been responders and compliant.

The superior relapse prevention finding over the long term agrees with the literature. Rosenheck and colleagues^27^ followed patients on clozapine (*n* = 122) or another antipsychotic (*n* = 123) for a year, with clinical assessments at weeks 6, 12, 26 and 52. They found that although clozapine invariably showed greater improvement in symptoms in all assessments, it was at 1 year that the quality of life difference was greatest. Alessi-Severini *et al*^28^ reported that of 74 patients on clozapine therapy in Manitoba, 60% have been on clozapine for 5 years, and had fewer relapses compared with the pre-clozapine period.

So far, the largest observational study of clozapine in children and youth is by Schneider and colleagues in Denmark, and it gave indirect evidence about clozapine's efficacy based on its continued use after 6 months and shorter hospital stays.^29^ In three randomised controlled trials, clozapine was compared head-to-head with another antipsychotic,^30^ and clozapine showed superior efficacy overall. Clozapine might have clinical use beyond the treatment of psychosis and bipolar disorder in the management of behavioural disorders including self-harm or aggression associated with ADHD, ODD and autism spectrum disorder (ASD). One study suggested that clozapine may be superior to treatment with risperidone in the management of aggression in children and adolescents with conduct disorder.^31^ More studies on the use of clozapine for behavioural disorders in children are needed.

Three implications for the clozapine treatment are suggested by the findings. First, the benefit of clozapine may outweigh its adverse effects in certain patients and should probably be prescribed more often for cases that do not respond to other anti-psychotics first. Fewer than 1% of both adult and child/youth patients were hospitalised for neutropenia, the most serious side effect. Pneumonia and constipation incidents were prominent adverse events in adults and may require clinical management. Despite a favourable benefit-risk balance for clozapine, only 10–20% of eligible patients are prescribed clozapine in the USA^32^ and the same is true in Canada.^33,34^ In Québec, less than 7% of schizophrenia patients received clozapine for 6 months or more in 2004,^33^ a figure that would ideally be closer to the prevalence of treatment-resistant schizophrenia, which is about 33%.^34^ Peters and colleagues reported that over a 5-year period, schizophrenia patients are on polypharmacy for 4 months, but only 17 days on clozapine.^35^ In Manitoba, the median length of time to clozapine initiation was 8 years.^28^

Second, health systems should provide support for logistical requirements associated with clozapine treatment. It is argued that underutilisation of clozapine in Japan is related to requirements for weekly blood tests for the first 26 weeks and 24/7 availability of a haematologist.^36^ This contrasts with the shorter requirement of 18 weeks of monitoring in Denmark.^37^ Third, some physicians’ attitudes and competence are a barrier to prescribing. Physicians believe that patients are unlikely to agree to blood testing and unlikely to adhere to treatment.^38^ A major barrier to appropriately prescribing clozapine is prescriber fear,^39^ but an education initiative in New York State increased clozapine prescribing.^40^

The main strength of this study is the long follow-up period and the use of recurrent events survival analysis. Had single-failure survival analysis been used instead, clozapine's benefit among adults in the long run might have been obscured because this technique considers only the time to the first event. It also has several limitations. There were no clinical variables such as age of illness onset, severity of illness and how soon treatment was initiated due to limitations of administrative data on hospitalisations – that is, they do not contain clinician assessments of patient function or validated rating scales. As such, it was not possible to tell how well patients functioned independent of relapse. This also applies to adverse events: without the benefit of clinical notes, it was not possible to tell if these were caused by clozapine (or other drugs) and what other conditions may have contributed to the hospitalisation. The medication data were not ideal because the number of days’ supply of medications was fixed at one month. The study was restricted to three provinces because these provinces enabled a linkage of hospitalisations and pharmacy redemption. Unfortunately, not all the hospitals in these provinces are required to submit all emergency and inpatient admissions to CIHI. It was not thought that this limitation favours one drug group over another.

Clozapine was associated with lower relapse overall, but this was accompanied by higher adverse events for adults. For children/youth, clozapine was associated with lower relapse all throughout and had no difference in adverse events compared with other antipsychotics.

## Supporting information

Balbuena et al. supplementary material 1 Balbuena et al. supplementary material

Balbuena et al. supplementary material 2 Balbuena et al. supplementary material

Balbuena et al. supplementary material 3 Balbuena et al. supplementary material

Balbuena et al. supplementary material 4 Balbuena et al. supplementary material

## Data Availability

The data that support the findings of this study are available from the Canadian Institute of Health Information. Restrictions apply to the availability of these data, which were used under licence for this study. Data are available from the authors with the permission of the Canadian Institute of Health Information, which can be obtained by writing to help@cihi.ca.

## Analytic code availability

The code used for implementing (reproducing) the analyses are publicly available at https://github.com/lloydxeno/antipsychotics and at https://github.com/sjhalayka/dad_nacrs.
