# Supplementary material for: Clozapine, relapse, and adverse events: a 10-year electronic cohort study in Canada
Source: Br J Psychiatry. 2024 Dec;225(6):572–8. doi: 10.1192/bjp.2024.140 (PMC11669471; doi:10.1192/bjp.2024.140)
Supplement: Balbuena et al. supplementary material 1 — Balbuena et al. supplementary material [file S0007125024001405sup001.docx]

**Supplementary Table 1: Royston-Parmar Recurrent Events Model of Mental Health Relapse in Adults from Three Canadian Provinces.**

| **Main effects** | **Hazard Ratio** | **Robust S.E.** | **z** | **95% CI** | |
| --- | --- | --- | --- | --- | --- |
| Clozapine | 0.86 | 0.018 | -7.33 | 0.83 | 0.90 |
| Age | 1.01 | 0.000 | 10.83 | 1.00 | 1.01 |
| Female | 1.01 | 0.016 | 0.83 | 0.98 | 1.04 |
| Rural/Other | 0.96 | 0.020 | -1.91 | 0.92 | 1.00 |
| **Time** | **Coefficient** | **Robust S.E.** | **z** | **95% CI** | |
| _spline1 | -15.74 | 0.141 | -111.76 | -16.01 | -15.46 |
| _spline2 | 3.14 | 0.059 | 53.63 | 3.02 | 3.25 |
| _spline3 | -1.33 | 0.027 | -48.86 | -1.39 | -1.28 |
| _spline4 | -0.88 | 0.026 | -34.35 | -0.93 | -0.83 |
| _spline5 | -0.52 | 0.034 | -15.06 | -0.58 | -0.45 |
| **Interactions with Time** |  |  |  |  |  |
| clozapine#c._spline_tvc1 | 2.11 | 0.168 | 12.56 | 1.78 | 2.44 |
| clozapine#c._spline_tvc2 | 0.15 | 0.077 | 1.88 | -0.01 | 0.30 |
| clozapine#c._spline_tvc3 | 0.14 | 0.025 | 5.61 | 0.09 | 0.19 |
| clozapine#c._spline_tvc4 | 0.12 | 0.022 | 5.18 | 0.07 | 0.16 |
| clozapine#c._spline_tvc5 | 0.07 | 0.028 | 2.68 | 0.02 | 0.13 |
| c.age#c._spline_tvc1 | -0.01 | 0.003 | -4.67 | -0.02 | -0.01 |
| c.age#c._spline_tvc2 | 0.00 | 0.001 | -0.06 | 0.00 | 0.00 |
| c.age#c._spline_tvc3 | 0.00 | 0.001 | -6.40 | 0.00 | 0.00 |
| c.age#c._spline_tvc4 | 0.00 | 0.000 | -9.83 | -0.01 | 0.00 |
| c.age#c._spline_tvc5 | -0.01 | 0.001 | -8.12 | -0.01 | 0.00 |
| female#c._spline_tvc1 | -0.24 | 0.093 | -2.59 | -0.42 | -0.06 |
| female#c._spline_tvc2 | -0.01 | 0.039 | -0.37 | -0.09 | 0.06 |
| female#c._spline_tvc3 | 0.03 | 0.017 | 1.65 | -0.01 | 0.06 |
| female#c._spline_tvc4 | 0.03 | 0.016 | 2.11 | 0.00 | 0.07 |
| female#c._spline_tvc5 | 0.03 | 0.021 | 1.18 | -0.02 | 0.07 |
| rural_unk#c._spline_tvc1 | 0.15 | 0.123 | 1.24 | -0.09 | 0.39 |
| rural_unk#c._spline_tvc2 | -0.16 | 0.048 | -3.30 | -0.25 | -0.06 |
| rural_unk#c._spline_tvc3 | 0.07 | 0.022 | 3.16 | 0.03 | 0.11 |
| rural_unk#c._spline_tvc4 | 0.05 | 0.021 | 2.30 | 0.01 | 0.09 |
| rural_unk#c._spline_tvc5 | 0.05 | 0.028 | 1.77 | -0.01 | 0.10 |
| _cons | 1.65 | 0.025 | 66.28 | 1.60 | 1.70 |
