# Supplementary material for: Clozapine, relapse, and adverse events: a 10-year electronic cohort study in Canada
Source: Br J Psychiatry. 2024 Dec;225(6):572–8. doi: 10.1192/bjp.2024.140 (PMC11669471; doi:10.1192/bjp.2024.140)
Supplement: Balbuena et al. supplementary material 2 — Balbuena et al. supplementary material [file S0007125024001405sup002.docx]

**Supplementary Table 2: Royston-Parmar Recurrent Events Model of Adverse Events in Adults from Three Canadian Provinces.**

| **Main effects** | **Hazard Ratio** | **Robust S.E.** | **z** | **95% CI** | |
| --- | --- | --- | --- | --- | --- |
| Clozapine | 1.34 | 0.09 | 4.33 | 1.18 | 1.54 |
| Age | 1.05 | 0.00 | 30.52 | 1.04 | 1.05 |
| Female | 0.85 | 0.03 | -4.11 | 0.79 | 0.92 |
| Rural/Other | 0.90 | 0.04 | -2.13 | 0.82 | 0.99 |
| **Time** | **Coefficient** | **Robust S.E.** | **z** | **95% CI** | |
| _spline1 | -12.42 | 1.07 | -11.58 | -14.52 | -10.32 |
| _spline2 | 1.62 | 0.45 | 3.64 | 0.75 | 2.50 |
| _spline3 | -1.24 | 0.11 | -10.83 | -1.46 | -1.01 |
| _spline4 | -0.80 | 0.10 | -8.45 | -0.99 | -0.62 |
| _spline5 | -0.40 | 0.14 | -2.93 | -0.67 | -0.13 |
| **Interactions with Time** |  |  |  |  |  |
| clozapine#c._spline_tvc1 | -2.55 | 1.89 | -1.35 | -6.25 | 1.14 |
| clozapine#c._spline_tvc2 | 2.60 | 0.97 | 2.69 | 0.71 | 4.50 |
| clozapine#c._spline_tvc3 | -0.04 | 0.11 | -0.32 | -0.25 | 0.18 |
| clozapine#c._spline_tvc4 | 0.05 | 0.08 | 0.66 | -0.11 | 0.22 |
| clozapine#c._spline_tvc5 | -0.01 | 0.10 | -0.11 | -0.22 | 0.19 |
| c.age#c._spline_tvc1 | -0.17 | 0.02 | -6.97 | -0.22 | -0.13 |
| c.age#c._spline_tvc2 | 0.04 | 0.01 | 3.61 | 0.02 | 0.07 |
| c.age#c._spline_tvc3 | -0.01 | 0.00 | -4.41 | -0.01 | 0.00 |
| c.age#c._spline_tvc4 | -0.01 | 0.00 | -4.06 | -0.01 | 0.00 |
| c.age#c._spline_tvc5 | -0.01 | 0.00 | -3.28 | -0.01 | 0.00 |
| _cons | -3.34 | 0.09 | -38.93 | -3.50 | -3.17 |
