# Supplementary material for: Clozapine, relapse, and adverse events: a 10-year electronic cohort study in Canada
Source: Br J Psychiatry. 2024 Dec;225(6):572–8. doi: 10.1192/bjp.2024.140 (PMC11669471; doi:10.1192/bjp.2024.140)
Supplement: Balbuena et al. supplementary material 3 — Balbuena et al. supplementary material [file S0007125024001405sup003.docx]

**Supplementary Table 3: Royston-Parmar Recurrent Events Model of Mental Health Relapse in Children/Youth from Three Canadian Provinces.**

| **Main effects** | **Hazard Ratio** | **Robust S.E.** | **z** | **95% CI** | |
| --- | --- | --- | --- | --- | --- |
|  |  |  |  |  |  |
| Clozapine | 0.62 | 0.07 | -4.03 | 0.49 | 0.78 |
| Age | 1.04 | 0.01 | 3.60 | 1.02 | 1.07 |
| Female | 1.01 | 0.07 | 0.12 | 0.88 | 1.15 |
| Rural/Other | 1.06 | 0.09 | 0.69 | 0.90 | 1.25 |
| **Time** | **Coefficient** | **Robust S.E.** | **z** | **95% CI** | |
| _spline1 | -16.43 | 0.30 | -55.47 | -17.01 | -15.85 |
| _spline2 | 3.34 | 0.13 | 26.40 | 3.09 | 3.59 |
| _spline3 | -1.62 | 0.06 | -28.14 | -1.73 | -1.50 |
| _spline4 | -1.14 | 0.05 | -21.68 | -1.24 | -1.03 |
| _spline5 | -0.78 | 0.08 | -9.73 | -0.94 | -0.63 |
| _cons | 1.15 | 0.21 | 5.40 | 0.73 | 1.57 |
