# Supplementary material for: Clozapine, relapse, and adverse events: a 10-year electronic cohort study in Canada
Source: Br J Psychiatry. 2024 Dec;225(6):572–8. doi: 10.1192/bjp.2024.140 (PMC11669471; doi:10.1192/bjp.2024.140)
Supplement: Balbuena et al. supplementary material 4 — Balbuena et al. supplementary material [file S0007125024001405sup004.docx]

**Supplementary Table 4: Royston-Parmar Recurrent Events Model of Adverse Events in Children/Youth from Three Canadian Provinces.**

| **Main effects** | **Hazard Ratio** | **Robust S.E.** | **z** | **95% CI** | |
| --- | --- | --- | --- | --- | --- |
|  |  |  |  |  |  |
| Clozapine | 0.99 | 0.46 | -0.02 | 0.40 | 2.47 |
| Age | 1.06 | 0.04 | 1.35 | 0.98 | 1.14 |
| Female | 1.22 | 0.31 | 0.80 | 0.75 | 2.00 |
| Rural/Other | 1.31 | 0.41 | 0.87 | 0.71 | 2.41 |
| **Time** | **Coefficient** | **Robust S.E.** | **z** | **95% CI** | |
| _spline1 | 1.12 | 0.12 | 9.72 | 0.90 | 1.35 |
| _cons | -8.48 | 0.95 | -8.94 | -10.34 | -6.62 |
